# 642. The impact of novel E1:M407I mutation on replicative fitness of the Chikungunya virus ECSA-IOL strain during infection of mammalian and mosquito cells,

**DOI:** 10.1093/ofid/ofad500.706

**Published:** 2023-11-27

**Authors:** Bilal A Khan, Saif Ullah, Saeed Khan

**Affiliations:** Dow University of Health Sciences, Karachi, Sindh, Pakistan; University of Karachi, Karachi, Sindh, Pakistan; Dow University of Health Sciences, Karachi, Sindh, Pakistan

## Abstract

**Background:**

Chikungunya virus (CHIKV) is an Alpha virus which is transmitted by Aedes genus of mosquitoes. It causes febrile illness with severe arthralgia, myalgia and rashes. Since 2000, the re-emergence of this pathogen have made it a global health security threat as it has caused severe form of diseases with epidemics of large magnitude and also spread to European and American continent. Studies have identified different point mutations in E1 and E2 region associated with virulence and infectivity, which is behind these severe epidemics. Pakistan reported first CHIKV outbreak with more than 30,000 infections from its largest city in 2016 which is already endemic for Dengue virus (DENV).

**Methods:**

32 CHIKV PCR-positive serum samples were collected from molecular pathology section of DUHS followed by RNA extraction and amplifcation of E1 region by PCR. The amplified products were prurified and sanger sequenced by BigDye termination method. Mutation analyses & Bayesian phylogeny was done by bioinformatic tools. CHIKV E1:M407I mutation was cloned in to CHIKV wild type infectious cDNA clone plasmid (SL-CK-1) by site directed mutagenesis (SDS). In vitro viral RNA was synthesized after plasmid linearization, which was electroporated into VERO-CCL-81 cells. M407I mutant virus was recovered from the culture, titrated by plaque assay and sanger sequenced to confirmed the M407I mutation. Viral competition assay was performed in mammalian and mosquitoes cell lines to determine the relative replicative fitness of mutant CHIKV virus over wild type.

**Results:**

Sequence analysis revealed 7 unique mutations in E1 including one mutation (M407I) in more than half of the sequenced samples. Phylogenetic analyses revealed this outbreak was caused by CHIKV-IOL genotype with possible inception from India. Virus competition assay showed relative replicative fitness for CHIKV E1:M407I mutant in Vero-CLL-81 and AAG2 cells have greater advantage while in C6/36, it has slight advantage over wild type virus.

**Conclusion:**

This study reports a novel mutations in E1 protein of CHIKV with fitness advantage over wild type strain. Phylogeny revealed the outbreak was caused by A226-IOL strain with possible origin from ancestral strain of Indian outbreak.

**Disclosures:**

**All Authors**: No reported disclosures

